# Recent biological and pharmacological activities of naringenin in clinical studies

**DOI:** 10.17179/excli2026-9460

**Published:** 2026-06-29

**Authors:** Fares Aljasmi, Cijo George Vazhappilly

**Affiliations:** 1 Department of Biotechnology, American University of Ras Al Khaimah, Ras Al Khaimah, United Arab Emirates

## ⁯

Naringenin is a dietary flavanone belonging to the flavonoid class of polyphenols (Polo-Castellano et al., 2024[4]). As a flavanone enriched in citrus matrices, dietary naringenin exposure is predominantly encountered in grapefruit, pummelo, oranges, and lemons (Shin and Shin, 2024[6]). In these citrus foods, naringenin occurs mainly as glycosylated flavanone derivatives, particularly naringin (naringenin-7-O-neohesperidoside) and prunin (naringenin-7-O-glucoside), with free aglycone present at lower levels (Jeffries et al., 2025[3]; Hallikeri et al., 2026[1]). Following ingestion, naringenin glycosides undergo intestinal cleavage to release the aglycone, which is subsequently subjected to phase II metabolism, yielding circulating and urinary naringenin glucuronide and sulfate conjugates (Serra et al., 2025[5]).

Across experimental models, naringenin has been linked to a multi-pathway profile that includes inflammatory signaling, antioxidant defense, and tumor/vascular remodeling. In a dextran sulfate sodium (DSS)-driven colitis and intestinal fibrosis model, naringenin was associated with redox defense patterns consistent with Nrf2/Keap1 modulation, and shifts in autophagy-related signaling coordinated through AMPK with downstream Akt/mTOR regulation (Hassan and Aubel, 2025[2]). In a macrophage inflammation model, naringenin suppressed pro-inflammatory cytokine expression through MT1G induction, accompanied by inhibition of NF-κB activation (Yang et al., 2024[8]). In malignant melanoma models, naringenin suppressed ERK1/2 and JNK MAPK signaling, promoted caspase-3-associated apoptosis in tumor cells, and inhibited angiogenesis (Tripathi et al., 2025[7]). Collectively, these mechanistic observations support biological plausibility and provide context for a recent shift toward clinical studies. Here, we summarize recent human and clinical evidence evaluating dietary exposure to naringenin and its circulating or urinary metabolites, as outlined in Supplementary Table 1.

## Declaration

### Acknowledgments

The authors thank the American University of Ras Al Khaimah (AURAK) for the support to prepare this manuscript.

### Conflict of interest

The authors declare no conflict of interest.

### Artificial Intelligence (AI) - assisted technology

The authors did not use any artificial intelligence-based technologies for the preparation of this manuscript.

## Supplementary Material

Supplementary information
