# Supplementary material for: Recent biological and pharmacological activities of naringenin in clinical studies
Source: EXCLI J. 2026 Jun 29;25:972–3. doi: 10.17179/excli2026-9460 (PMC13369879; doi:10.17179/excli2026-9460)
Supplement: Supplementary information [file EXCLI-25-972-s-001.pdf]

EXCLI Journal 2026;25:972-973 – ISSN 1611-2156
Received: April 03, 2026, accepted: May 23, 2026, published: June 29, 2026

Supplementary information to:

Letter to the editor:

RECENT BIOLOGICAL AND PHARMACOLOGICAL ACTIVITIES OF NARINGENIN IN CLINICAL STUDIES

Fares Aljasmi, Cijo George Vazhappilly*

Department of Biotechnology, American University of Ras Al Khaimah, Ras Al Khaimah, United Arab Emirates

* **Corresponding author:** Cijo George Vazhappilly, Department of Biotechnology, American University of Ras Al Khaimah, Ras Al Khaimah, United Arab Emirates; Tel.: +971-7-246-8842; E-mail: cijo.vazhappilly@aurak.ac.ae

https://dx.doi.org/10.17179/excli2026-9460

This is an Open Access article distributed under the terms of the Creative Commons Attribution License (https://creativecommons.org/licenses/by/4.0/).

Supplementary Table 1: Recent pharmacological activities of naringenin in clinical studies

Key Evidence	Reference
A cross-sectional exposome-based analysis using NHANES datasets (n = 10,018; 9,033 without kidney stones and 985 with kidney stones) evaluated associations between dietary flavonoid intake and kidney stone development risk. A nonlinear J-shaped relationship was observed with the lowest estimated risk occurring at 32.23 mg/day (OR 0.70, 95 % CI 0.58–0.84). Within the flavanone subgroup, naringenin intake was negatively associated with kidney stone risk, suggesting potential protective dietary effects of citrus-derived flavanones.	Gong et al., 2026
In a 6-week randomized, open-label crossover trial (n = 20 adults aged 30–60; control n = 10, MetS n = 10), participants consumed 330 mL/day of three different beer types to profile beer-derived urinary polyphenolic metabolites in adults with and without MetS. Overall, 20 phenolic metabolites changed after the intervention (12 increased, 8 decreased). Measurable naringenin and its Phase II conjugate, naringenin glucuronide, increased in controls and decreased or remained almost unaltered in the MetS group, suggesting altered microbiome catabolism, intestinal absorption, or phase II conjugation. Additionally, urinary naringenin metabolites correlated negatively with triglycerides and insulin, and positively with HDL-cholesterol. Phenotype-dependent biotransformation of beer-derived polyphenols, including naringenin, may relate to oxidative stress and inflammation pathways.	Hinojosa-Nogueira et al., 2025

EXCLI Journal 2026;25:972-973 – ISSN 1611-2156
Received: April 03, 2026, accepted: May 23, 2026, published: June 29, 2026

Key Evidence	Reference
A cross-sectional, observational study using NHANES data (n = 1,854 participants with diabetes; 1,481 without DR and 373 with DR) examined whether estimated dietary naringenin intake was associated with the presence of DR. Higher naringenin intake (high category > 9.888 mg/day) was associated with lower odds of DR, including in the fully adjusted model (OR 0.38, 95 % CI 0.16–0.89), with a stronger inverse association among participants with a diabetes duration of > 10 years (OR 0.26, 95 % CI 0.08–0.82). Supportive in vitro experiments in human retinal microvascular endothelial cells under high glucose conditions (30 mmol/L D-glucose) suggested that naringenin (40 µM) reduced hyperglycemia-induced injury by inhibiting apoptosis. Apoptosis was reduced from 25.58 % under high glucose to 8.17 % with naringenin co-treatment, compared with 5.02 % in normal glucose controls. This anti-apoptotic effect was accompanied by increased Bcl-2, alongside decreased Bax and cleaved caspase-3.	Chen et al., 2025
A cross-sectional, observational study using NHANES data (n = 12,134 adults aged ≥ 20; 4,450 with hypertension) evaluated associations between estimated dietary flavonoid intake and hypertension risk. Higher estimated naringenin intake (mean ~3.79 mg/day) was inversely associated with hypertension, including in the fully adjusted model (OR 0.946, 95 % CI 0.909–0.984). A SHAP-based model interpretation ranked naringenin (alongside quercetin) as the 2 most important flavonoids contributing to a lower predicted hypertension risk.	Wu et al., 2025
A cross-sectional, observational study using NHANES data (n = 2,581 participants aged ≥ 18; 555 diagnosed with MASLD) examined associations between energy-adjusted dietary flavonoid intake and MASLD. In a 29-flavonoid WQS mixture model, naringenin was among the highest-weight contributors to the protective flavonoid index (24.0 %). In single-compound analyses, higher naringenin intake (top versus bottom tertile) was significantly associated with reduced odds of MASLD (OR 0.38, 95 % CI 0.20–0.71), and energy-adjusted naringenin intake was lower in participants with MASLD than in those without MASLD (2.88 vs 3.31 mg/day).	Wang et al., 2025
In a 36-week randomized controlled trial (n = 80 older adults aged 60–75), participants received 400 mg/day of citrus peel extract containing ~3.0 mg naringenin, examining SCD. The citrus peel group lacked statistically significant cognitive improvement versus the placebo group, with increasing RBANS scores over time in both groups (103.6 ± 8.6 to 108.2 ± 8.6, mean change 4.63 ± 1.57 in the citrus peel group; 101.8 ± 8.6 to 107.4 ± 8.6, mean change 5.63 ± 1.57 in placebo). The biomarker subset showed increased pro-inflammatory IL-8 levels over time, which were not reduced by the intervention.	Galluzzi et al., 2024
A prospective, observational study using EPIC cohort subsamples (n = 761 participants; 194 with 5-year body weight loss, 274 with maintenance, and 293 with gain) examined associations between 5-year body weight change from baseline and plasma concentrations of 36 polyphenols. While several plasma polyphenols showed suggestive associations with weight maintenance or loss, naringenin (median plasma concentration of ~3.70 nmol/L) showed only a borderline association with weight loss versus maintenance (OR 1.1, 95 % CI 1.0–1.2) that did not survive FDR correction.	Gil-Lespinard et al., 2024

EXCLI Journal 2026;25:972-973 – ISSN 1611-2156
Received: April 03, 2026, accepted: May 23, 2026, published: June 29, 2026

Key Evidence	Reference
A cross-sectional, observational study using NHANES data (n = 5,970 participants aged 6–18; 2,463 with overweight/obesity and 1,294 with central obesity) assessed associations between adiposity outcomes and dietary flavanone intake in children and adolescents. Higher naringenin intake (> 0.32 mg/1000 kcal) was inversely associated with overweight/obesity (OR 0.76, 95 % CI 0.63–0.92) and central obesity (OR 0.69, 95 % CI 0.55–0.86), relative to no intake. Sex- and age-stratified analyses suggested stronger inverse associations for central obesity among 12–18-year-olds (OR 0.69, 95 % CI 0.50–0.93) and males (OR 0.67, 95 % CI 0.51–0.89).	Liu et al., 2024
An ex vivo study using murine and human PCLS assessed naringenin's antifibrotic activity in culture-induced early fibrogenesis (mPCLS and hhPCLS) and established fibrosis (chPCLS). In hhPCLS and chPCLS cultures, 300 µM naringenin reduced ATP/protein (cytotoxicity), therefore, antifibrotic effects were interpreted at non-cytotoxic concentrations (≤ 200 µM). Naringenin produced dose-dependent suppression of collagen type I production, suppressing COL1A1 expression (up to ~64 %) and secreted PCOL1A1 (up to ~78 %) in chPCLS. This antifibrotic activity was further accompanied by reduced expression of myofibroblast activation/remodeling markers ACTA2 (up to ~35 %) and SERPINE1 (up to ~60 %) in chPCLS. Naringenin showed limited effects on inflammatory transcripts but reduced IL-1β cytokine release, while IL-6 was not consistently altered.	Luo et al., 2024
A metabolomics analysis of plasma samples from a previously conducted two-month intervention trial (n = 140 overweight adults) evaluated daily consumption of a citrus-maqui drink sweetened with sucrose, sucralose, or stevia, examining sex differences in circulating polyphenol metabolite profiles. Flavanone metabolites were detectable in plasma and showed time-associated changes, with naringenin glucoside identified as among the two flavanone metabolites showing a significant time effect by ANOVA ($p = 1.8 \times 10^{-4}$). Pairwise analyses suggested time-sex and time-sweetener trends not consistently supported by ANOVA. This includes a greater time-related increase in naringenin glucoside levels in women under certain sweetener conditions relative to baseline.	Hernández-Prieto et al., 2023
An acute, crossover trial (n = 11 healthy adults aged 18–40) evaluated 400 g of spray-dried versus freeze-dried grapefruit juice consumption on two test days separated by a 15-day washout. It examined circulating bioactive compound levels including serum aglycone naringenin under fasting conditions and at 4 hours post-intake. Mean serum naringenin concentrations increased from 4.2 ± 0.8 mg/L at fasting to 5.2 ± 0.4 mg/L after freeze-dried juice and 4.6 ± 0.9 mg/L after spray-dried juice, corresponding to an average relative increase of ~28 %. This was accompanied by substantial inter-individual variability (relative change range: -35 % to 106 %; seven naringenin measurements showed CV > 15 %) and no significant differences between processing methods. Additionally, naringenin did not show a significant correlation with serum radical scavenging ability.	Camacho et al., 2023

Key Evidence	Reference
A longitudinal, observational study using NHANES mortality-linked data (n = 14,029 participants aged ≥ 18; 405 cancer deaths) assessed the association between dietary flavonoid intake and cancer mortality over a median follow-up of 117 months. Univariable (hazard ratio [HR] 0.97, 95 % CI 0.95–0.99) and multivariable (HR 0.97, 95 % CI 0.95–1.00) Cox models suggested that higher naringenin intake was inversely associated with cancer mortality, with a higher estimated survival probability for participants over the 90th percentile of naringenin intake. Stratified analyses suggested the inverse association was more evident among participants with hyperlipidemia (HR 0.95, 95 % CI 0.91–0.99), Black participants (HR 0.93, 95 % CI 0.88–0.98), and those aged > 50 (HR 0.96, 95 % CI 0.93–0.99). Descriptively, most participants who died from cancer reported lower naringenin intake (mean ~2.62 ± 0.36 mg/day) than those who remained alive (mean ~3.40 ± 0.16 mg/day).	Zhou et al., 2023
A cross-sectional study using baseline data from the PREDIMED-Plus randomized controlled trial (n = 266 participants with MetS, aged 55–75) examined associations between urinary phase II flavonoid metabolites, liver enzymes, and inflammatory scores, as well as the association between the consumption of polyphenol-rich foods and urinary flavonoid metabolite concentrations. Naringenin conjugates were naringenin-4′-glucuronide (naringenin 4′-GlcUA) and naringenin-7′-glucuronide (naringenin 7′-GlcUA), with mean urinary concentrations of 18.0 ± 38.3 and 11.0 ± 25.9 nmol/g creatinine, respectively. These showed significant inverse associations with gamma-glutamyl transferase (GGT) levels (B per 1 SD = −0.15, 95 % CI −0.26 to −0.03 for naringenin 4′-GlcUA; B per 1 SD = −0.12, 95 % CI −0.24 to −0.01 for naringenin 7′-GlcUA), consistent with healthier liver enzyme profiles. Higher naringenin 7′-GlcUA excretion showed borderline inverse associations with lower AISI and SII scores (B per 1 SD = −0.14, 95 % CI −0.27 to −0.02 for both), but these did not survive FDR correction. Spearman analyses suggested that naringenin conjugates reflect dietary intake, showing a moderate positive association with higher citrus consumption (rho = 0.286 for naringenin 4′-GlcUA; rho = 0.348 for naringenin 7′-GlcUA) and weaker correlations with olives, walnuts/nuts, and whole-grain bread.	Bullón-Vela et al., 2023

Abbreviations: NHANES, National Health and Nutrition Examination Survey; **OR**, odds ratio; **CI**, confidence interval; **SCD**, subjective cognitive decline; **RBANS**, Repeatable Battery for the Assessment of Neuropsychological Status; **IL-8**, interleukin-8; **MetS**, metabolic syndrome; **HDL**, high-density lipoprotein; **ANOVA**, analysis of variance; **CV**, coefficient of variation; **DR**, diabetic retinopathy; **Bcl-2**, B-cell lymphoma 2; **Bax**, BCL2-associated X protein; **EPIC**, European Prospective Investigation into Cancer and Nutrition; **FDR**, false discovery rate; **SHAP**, Shapley Additive exPlanations; **MASLD**, metabolic dysfunction-associated steatotic liver disease; **WQS**, weighted quantile sum; **HR**, hazard ratio; **GGT**, gamma-glutamyl transferase; **AISI**, Aggregate Index of Systemic Inflammation; **SII**, Systemic Inflammatory Index; **PCLS**, precision-cut liver slices; **mPCLS**, murine PCLS; **hhPCLS**, healthy human PCLS; **chPCLS**, cirrhotic human PCLS; **ATP**, adenosine triphosphate; **COL1A1**, collagen type I alpha 1 chain; **PCOL1A1**, procollagen type I alpha 1; **ACTA2**, actin alpha 2, smooth muscle; **SERPINE1**, serpin family E member 1; **IL-1β**, interleukin-1 beta; **IL-6**, interleukin-6; **rho**, Spearman correlation coefficient

Acknowledgments

The authors thank the American University of Ras Al Khaimah (AURAK) for the support to prepare this manuscript.

Conflict of interest

The authors declare no conflict of interest.

Artificial Intelligence (AI) – assisted technology

The authors did not use any artificial intelligence-based technologies for the preparation of this manuscript.

EXCLI Journal 2026;25:972-973 – ISSN 1611-2156
Received: April 03, 2026, accepted: May 23, 2026, published: June 29, 2026

REFERENCES

Bullón-Vela V, Xu Y, Razquin C, Abete I, Zulet MA, Martínez-González MA, et al. Health associations of liver enzymes and inflammatory scores with urinary citrus flavonoid metabolites. Food Funct. 2023;14:1011–23.

Camacho MDM, Martínez-Lahuerta JJ, García-Martínez E, Igual M, Martínez-Navarrete N. Bioavailability of Bioactive Compounds from Reconstituted Grapefruit Juice as Affected by the Obtention Process. Molecules. 2023;28:2904.

Chen Y, Cao Y, Zhu W, Huang Z. Exploration of the potential therapeutic benefits of naringenin against diabetic retinopathy through a National comprehensive cross-sectional study and in vitro experiments. Diabetol Metab Syndr. 2025;17:304.

Galluzzi S, Marizzoni M, Gatti E, Bonfiglio NS, Cattaneo A, Epifano F, et al. Citrus supplementation in subjective cognitive decline: results of a 36-week, randomized, placebo-controlled trial. Nutr J. 2024;23:135.

Gil-Lespinard M, Almanza-Aguilera E, Castañeda J, Guiñón-Fort D, Eriksen AK, Tjønneland A, et al. Plasma Concentration of 36 (Poly)phenols and Prospective Body Weight Change in Participants from the EPIC Cohort. Ann Nutr Metab. 2024;80:87–100.

Gong Y, Yang Y, Miao L, Zhong M, Xia Q, Chang J, et al. Flavonoid intake and kidney stones: An exposome-based cross-sectional study. J Nutr Biochem. 2026;147:110117.

Hernández-Prieto D, Fernández PS, Agulló V, García-Viguera C, Egea JA. Bioactive Compounds in Plasma as a Function of Sex and Sweetener Resulting from a Maqui-Lemon Beverage Consumption Using Statistical and Machine Learning Techniques. Int J Mol Sci. 2023;24:2140.

Hinojosa-Nogueira D, Díaz-Perdigones CM, García-López MJ, Marcos A, Portillo MP, Lamuela-Raventós RM, et al. Beer-Derived (Poly)phenol Metabolism in Individuals With and Without Metabolic Syndrome: A Comparative Dietary Intervention. Molecules. 2025;30:2932.

Liu Y, Liu Z, Wu N. Association between intake of flavanones and the overweight/obesity and central obesity in children and adolescents: a cross-sectional study from the NHANES database. Front Nutr. 2024;11:1430140.

Luo K, Geng Y, Oosterhuis D, de Meijer VE, Olinga P. Evaluating the antifibrotic potential of naringenin, asiatic acid, and icariin using murine and human precision-cut liver slices. Physiol Rep. 2024;12:e16136.

Wang C, Li M, Zhang J, Li H, Li Y, Huang S, et al. Associations of the Intake of Individual and Multiple Flavonoids with Metabolic Dysfunction Associated Steatotic Liver Disease in the United States. Nutrients. 2025;17:205.

Wu F, Wu Y, Chen Q, Yang X, Yu Z. Individual flavonoids intake and its association with hypertension in US adults: A population-based cross-sectional study. Medicine (Baltimore). 2025;104:e45553.

Zhou Y, Gu K, Zhou F. Dietary Flavonoid Intake and Cancer Mortality: A Population-Based Cohort Study. Nutrients. 2023;15:976.
